# LOW RISK CHEST PAIN FOLLOW UP: A CASE SERIES

**DOI:** 10.51894/001c.122820

**Published:** 2024-08-30

**Authors:** Nick Stiles, Virgina Labond

**Affiliations:** 1 Emergency Medicine Ascension Genesys

## 11

### INTRODUCTION

Chest pain is a common presenting complaint for patients seeking care in the Emergency Department. The HEART score is a validated pathway commonly used by Emergency Department Physicians to help determine which patients may be safe for discharge and outpatient follow up. We report a small case series of patients who were discharged from the Emergency Department with low-risk chest pain based on the HEART score and subsequently underwent telephone follow up to see if they were able to obtain timely follow up in the outpatient setting.

### METHODS

Patients were screened by a physician in triage or occasionally one already roomed in the ED and consented for participation. For those patients who consented, a chart review was performed to determine discharge diagnosis and HEART score at discharge. Those found to be appropriate candidates for the study following their ED workup and disposition were called back at 6 weeks following their visit and asked a brief series of questions regarding follow up and barriers to follow up.

### RESULTS

Phone call follow up was achieved in three patients. All three patients were able to follow up in the outpatient setting within six weeks following their Emergency Department visit. Only one patient had additional testing ordered. No barriers to follow up were identified.

### CONCLUSIONS

No strong conclusions regarding the frequency or ease of follow-up can be drawn from this small, observational case series. A larger study including more patients would be helpful in determining whether these results can be generalized to similar patient populations.

